# Supplementary material for: Mitochondrial sodium/calcium exchanger NCLX regulates glycolysis in astrocytes, impacting on cognitive performance
Source: J Neurochem. Author manuscript; Available in PMC 2023 Sep 5. (PMC10478152; doi:10.1111/jnc.15745)
Supplement: Supporting Information [file NIHMS1922642-supplement-Supporting_Information.pdf]

# Mitochondrial sodium/calcium exchanger NCLX regulates glycolysis in astrocytes, impacting on cognitive performance

João Victor Cabral-Costa, Carlos Vicente-Gutiérrez, Jesús Agulla, Rebeca Lapresa, John W. Elrod, Ángeles Almeida, Juan P. Bolaños, Alicia J. Kowaltowski

## Supporting information

[Fig2_Suppl1.avi]

**Figure 2 – Supplement 1. DMSO and CGP FURA-2 imaging representative videos.**

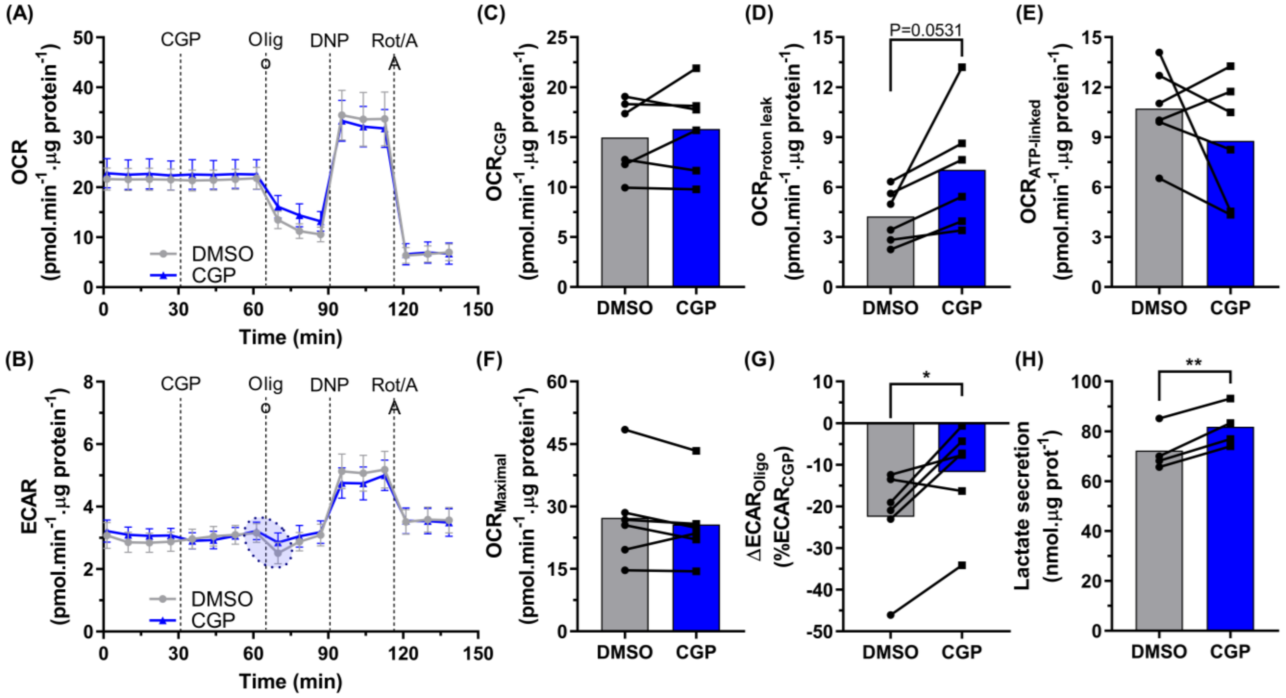


**Figure 3 –Supplement 1. C6 cells present increased lactate secretion upon NCLX inhibition.** C6 cells incubated with the NCLX inhibitor CGP-37157 (CGP) or DMSO had their oxygen consumption rate (OCR) and extracellular acidification rate (ECAR) monitored in a MitoStress Seahorse assay. Representative traces of (A) OCR and (B) ECAR from C6 cells acutely incubated with CGP, followed by oligomycin (oligo), 2,4-dinitrophenol (DNP), and rotenone plus antimycin A (Rot/AA) additions, average ± SEM; (C) CGP-induced, (D) proton leak-associated, (E) ATP-linked, and (F) maximal respirations; (G) ECAR variation after ATP synthase inhibition with oligomycin. Paired 2-way ANOVA followed by Holm-Šidak's post-hoc test, n = 6 independent experiments. (H) lactate secretion measured from C6 cells incubated with CGP or DMSO for 4 h. *$P < 0.05$, Student's t test, n = 4 independent experiments, mean and SD (A,B) or mean and paired measurements (C-H).

# Mitochondrial sodium/calcium exchanger NCLX regulates glycolysis in astrocytes, impacting on cognitive performance

João Victor Cabral-Costa, Carlos Vicente-Gutiérrez, Jesús Agulla, Rebeca Lapresa, John W. Elrod, Ángeles Almeida, Juan P. Bolaños, Alicia J. Kowaltowski

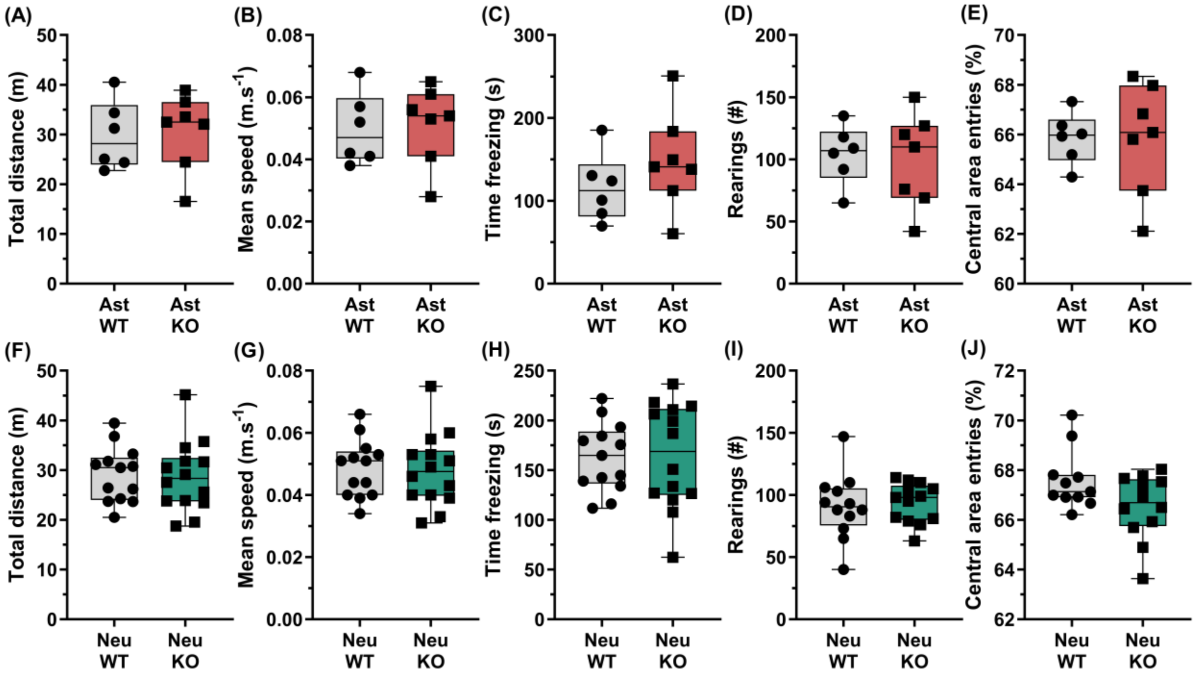


**Figure 5 – Supplement 1. Open Field test supplementary analyses.** Supplementary analyses from the Open Field test in mice with hippocampal astrocyte- (A-E) or neuronal-specific (F-J) NCLX deletion: (A,F) total distance; (B,G) mean speed; (C,H) total time in freezing behavior; (D,I) number of rearings; and (E,J) total entries in the central area. Not significant, unpaired Student's t test (A-C,F-H) or Mann-Whitney test (D,E,I,J), n = 6-14, box indicates upper and lower quartiles and the median (line), and whiskers represent min and max values.

[Fig5_Suppl2.avi]

**Figure 5 – Supplement 2. Open Field test – *In vivo* hippocampal astrocyte- and neuronal-specific NCLX deletion representative videos.**

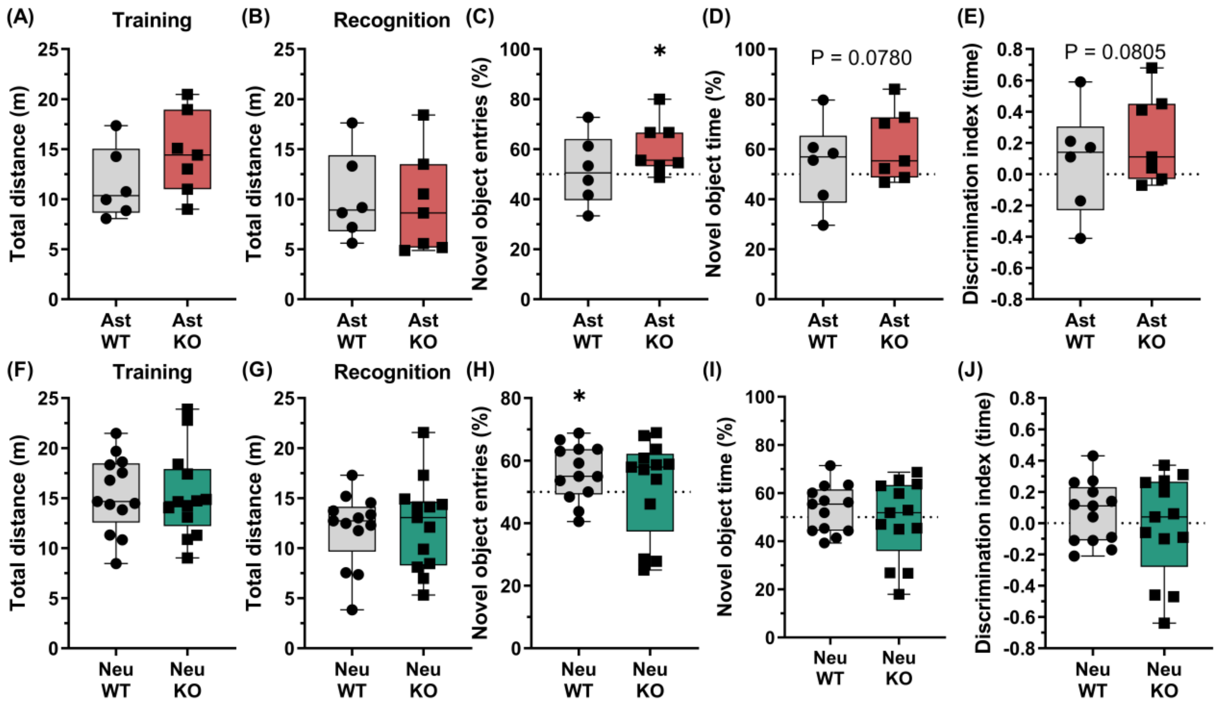


**Figure 5 – Supplement 3. Novel Object Recognition test supplementary analyses.** Supplementary analyses from the Novel Object Recognition test in mice with hippocampal astrocyte- (A-E) or neuronal-specific (F-J) NCLX deletion: (A,F) total distance in training and (B,G) recognition assay steps; (C,H) proportion of entries into and (D,I) time within the novel object area; and (E,J) discrimination index calculated from time in the novel and familiar object areas. *P < 0.05, unpaired Student's t test (A,B,D,E,F,G,H,I) or Mann-Whitney test (C,H), n = 6-14, box indicates upper and lower quartiles and the median (line), and whiskers represent min and max values.

[Fig5_Suppl4.avi]

**Figure 5 – Supplement 4. Novel Object Recognition test – *In vivo* hippocampal astrocyte- and neuronal-specific NCLX deletion representative videos.**

# Mitochondrial sodium/calcium exchanger NCLX regulates glycolysis in astrocytes, impacting on cognitive performance

João Victor Cabral-Costa, Carlos Vicente-Gutiérrez, Jesús Agulla, Rebeca Lapresa, John W. Elrod, Ángeles Almeida, Juan P. Bolaños, Alicia J. Kowaltowski

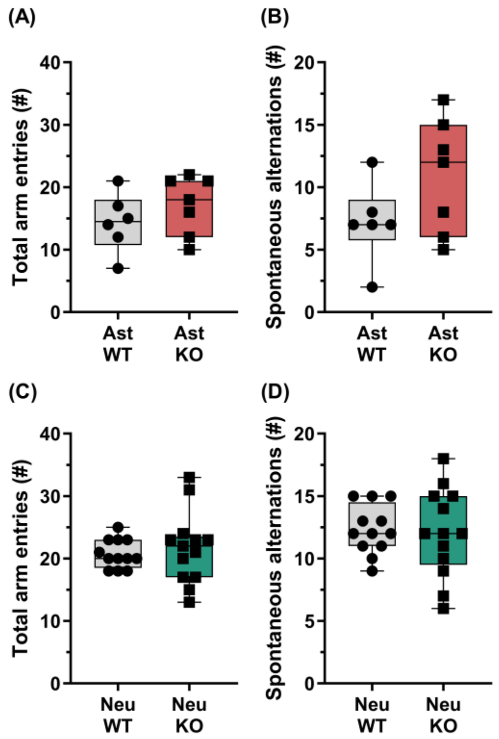


**Figure 5 – Supplement 5. Y-maze test supplementary analyses.** Supplementary analyses from the Y-maze test in mice with hippocampal astrocyte- (A,B) or neuronal-specific (C,D) NCLX deletion: (A,C) total number of arm entries; and (B,D) total number of spontaneous alternations. Not significant, Mann-Whitney test, n = 6-14, box indicates upper and lower quartiles and the median (line), and whiskers represent min and max values.

[Fig5_Suppl6.avi]

**Figure 5 – Supplement 6. Y-maze test – *In vivo* hippocampal astrocyte- and neuronal-specific NCLX deletion representative videos.**
